# Downregulation of Bmi1 in breast cancer stem cells suppresses tumor growth and proliferation

**DOI:** 10.18632/oncotarget.16317

**Published:** 2017-03-17

**Authors:** Mathangi Srinivasan, Dhruba J. Bharali, Thangirala Sudha, Maha Khedr, Ian Guest, Stewart Sell, Gennadi V. Glinsky, Shaker A. Mousa

**Affiliations:** ^1^ The Pharmaceutical Research Institute, Albany College of Pharmacy and Health Sciences, Rensselaer, NY, USA; ^2^ Division of Clinical Chemistry and Laboratory Medicine, Department of Clinical Pathology, Ain Shams University, Cairo, Egypt; ^3^ Wadsworth Center, New York State Department of Health, Albany, NY, USA; ^4^ Institute of Engineering in Medicine, University of California San Diego, La Jolla, CA, USA

**Keywords:** Bmi1, breast cancer stem cell, downregulation, PTC 209

## Abstract

Targeting cancer stem cells during initial treatment is important to reduce incidence of recurrent disease. Bmi1 has been associated with cancer stem cell self-renewal and aggressive disease. The purpose of this study was to determine the effects of downregulation of Bmi1 in breast cancer stem cells in order to target and eliminate the stem cell population in the tumor mass. Bmi1 was downregulated using two approaches in the mouse breast cancer stem cell line FMMC 419II—a small molecule inhibitor (PTC 209) and stable transfection with a Bmi1 shRNA plasmid. The functional effect of Bmi1 downregulation was tested *in vitro* and in *vivo*. Each approach led to decreased Bmi1 expression that correlated with an inhibition of cancer stem cell properties *in vitro* including cell cycle arrest and reduced mammosphere forming potential, and a decrease in tumor mass *in vivo* after either intra-tumoral or systemic nanoparticle-targeted delivery of anti-Bmi1. These results show that inhibiting Bmi1 expression in breast cancer stem cells could be important for the complete elimination of tumor and potentially preventing disease relapse.

## INTRODUCTION

Cancer stem cells (CSCs) are resistant to conventional radiotherapy and chemotherapy regimens, leading to high relapse rates and poor overall survival [1]. Therefore new therapeutic strategies specifically targeting these CSCs may overcome disease resistance and improve treatment efficacy.

CSCs are characterized by their ability to undergo self-renewal and are highly tumorigenic, with the capability to phenotypically recapitulate the primary tumor from which they have been derived [2]. In breast cancer, CSCs have been implicated in essential stages of tumor progression such as invasiveness and distant organ metastasis along with drug resistance [3, 4]. Identification of CSC-specific markers has aided in the isolation and characterization of the CSCs in many tumor models, for example, the CD44^+^CD24^−/low^Lin^−^ population of human breast cancers [5]. High ALDH1 (aldehyde dehydrogenase 1) activity has also been used as a functional marker to define the CSCs [6]. The phenotype in mouse breast CSCs is different from that of human. Mouse breast CSCs derived from MMTV-PyMT mice that develop spontaneous breast adenocarcinomas in female mice as early as 5 weeks have high expression of CD24 and CD49f [7]. CD49f (integrin subunit α6) is a marker for breast cancer progenitor cells, whereas CD24 is highly expressed on both CSCs and tumor transit amplifying cells [8, 9]. A cell line derived from the breast tumors in MMTV-PyMT mice, FMMC 419II, is highly enriched for breast CSCs using the characterization markers (Lin^−^CD24^high^CD49f^high^) and a single-cell tumor-initiation activity [7].

Multiple developmental pathways regulate CSC function and maintenance including the Notch, Hedgehog, and Wnt pathways [10]. Many molecules within these signal transduction pathways have been targeted to disrupt the CSC population [11]. One such molecule, Bmi1, is a Polycomb group (PcG) transcriptional repressor protein that silences gene expression by inducing chromatin modifications, hindering transcription factors and RNA polymerase binding [12]. Microarray analysis-defined 11-gene expression signature of the Bmi1 regulated stemness pathway identifies cancer patients with poor prognosis and increased likelihood of death from cancer [12]. Bmi1 is implicated in CSC self-renewal through regulation of genes important for cell cycle control and stem cell fate decisions, as well as regulation of survival genes and inhibition of cellular senescence in multiple cancer models [13, 14]. Bmi1 controls important stages in cancer progression including invasion and metastasis by modulating epithelial-mesenchymal transition (EMT) [15, 16] and drug resistance [17]. There is a statistically significant correlation between Bmi1 expression levels and poor prognosis and survival [18] as well as aggressiveness [19] in human breast cancer patients. Inhibiting expression of the *Bmi1* gene by stable transfection with shRNA to *Bmi1* also inhibits proliferation of FMMC 419II cells *in vitro* and blocks tumor initiation [7].

In this study, we use two parallel and independent approaches to inhibit Bmi1 in FMMC 419II breast CSCs: 1) PTC 209, a Bmi1-specific small molecule inhibitor and 2) shRNA to Bmi1. PTC 209 was discovered by high throughput screening of compounds utilizing the gene expression modulation by small molecules (GEMS) technology and shown to downregulate Bmi1 expression in colorectal cancer initiating cells [20]. PTC 209 was tested *in vitro* against biliary tract cancer and acute myeloid leukemia by other investigators and our group [21–23].

This is the first study directly assessing the effect of Bmi1 inhibition, using both molecular and pharmacological approaches, in a highly enriched population of CSCs *in vitro* and after transplantation into syngeneic fully immunocompetent animals.

We demonstrate that both PTC 209 treatment and stable transfection with a *Bmi1*-specific shRNA plasmid decrease Bmi1 mRNA and protein expression in the mammary CSCs and inhibit proliferation *in vitro* and tumor growth *in vivo* at relatively lower doses after orthotopic implantation into syngeneic fully immunocompetent host. Furthermore, nano-targeted delivery of PT 209 encapsulated into anti-CD49f poly (lactic-*co*-glycolic acid)poly (ethylene glycol) nanoparticles (PLGA-PEG NPs) resulted in a significant suppression of implanted mouse CSC into immunocompetent mice as compared to free PTC 209 or to PTC 209 encapsulated into PLGA-PEG NPs and without anti-CD49.

The application of nanotechnology in medicine offers unprecedented opportunities for addressing many of the current challenges in cancer therapy [24–26], in particular targeting stem cells to treat various kinds of cancers. NPs that are biodegradable and biocompatible are especially desirable for ease of *in vivo* preclinical and clinical utility [26, 27]. PLGA-based controlled release polymer has been utilized clinically, and its clinical safety and feasibility is well established [26–28]. PEG-functionalized NPs are important to enhance pharmacokinetics of these drugs [24]. Biodegradable PLGA-PEG NPs can be targeted for delivery of drugs along with potentially more sensitive diagnostic imaging options. As a proof of the concept, we have demonstrated our expertise in the field of imaging and targeted drug delivery [24, 26–33]. For this study we hypothesized that incorporation of PTC 209 into anti-CD49f PLGA-PEG NPs for targeted delivery will not only increase the accumulation of Bmi1 inhibitor PTC 209 into implanted breast CSC tumor and hence anti-cancer efficacy through active targeting, but will also enable improvement of its safety by using lower doses.

## RESULTS

### PTC 209 treatment or shRNA stable transfection decreases Bmi1 expression

**Figure 1:**
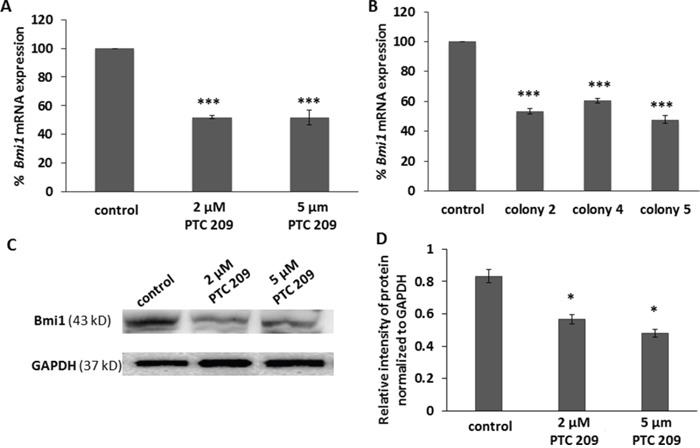
PTC 209 treatment and *Bmi1* shRNA transfection decreases *Bmi1* mRNA expression **(A)** Cells treated with PTC 209 and FMMC 419II cells stably transfected with *Bmi1* shRNA plasmid show a decrease in *Bmi1* mRNA expression. **(B)** Purified mRNA from the cells was reverse transcribed into cDNA and then analyzed for *Bmi1* mRNA expression with quantitative PCR using TaqMan gene expression assays. The fold difference in expression between control samples and the PTC 209 treated of the *Bmi1* shRNA transfected samples was calculated using the average of the Ct (threshold cycle) per group, relative to the expression of the internal control gene *GAPDH*. **(C)** FMMC cells were treated with PTC 209 (2 μM and 5 μM) for 24 hours and the expression of Bmi1 protein was detected with western blot. **(D)** PTC 209 treatment decreased Bmi1 protein expression in FMMC cells. Results are represented as mean ± S.E.M., *P <0.01, ***P <0.005.

Ma *et al*. have previously identified that the CD24^+^CD49f^+^ FMMC 419II cells isolated from the MMTV-PyMT tumors express Bmi1 at a significantly higher level than the other cell populations in these tumors that do not exhibit tumor initiation and other CSC-like properties [7]. Here, we observed that RT-PCR analysis of the CD24^+^CD49f^+^ FMMC 419II cells that were treated with various concentrations of the Bmi1 specific small molecule inhibitor PTC 209 show a significant decrease in *Bmi1* expression (Figure 1A), as does the analysis of cells after transfection with *Bmi1*-specific shRNAs (Figure 1B). For the stable transfection with inhibitory RNA, the FMMC 419II cells were transfected with a *Bmi1*-specific shRNA plasmid construct and colonies 2, 4, and 5 were obtained. RT-PCR analysis of these three colonies confirms that knockdown of *Bmi1* with shRNA transfection significantly decreases *Bmi1* mRNA expression. The significant decrease in expression of Bmi1 protein is seen with western blot analysis (Figure 1C and 1D).

### Inhibition of Bmi1 expression inhibits cell cycle progression and proliferation

**Figure 2:**
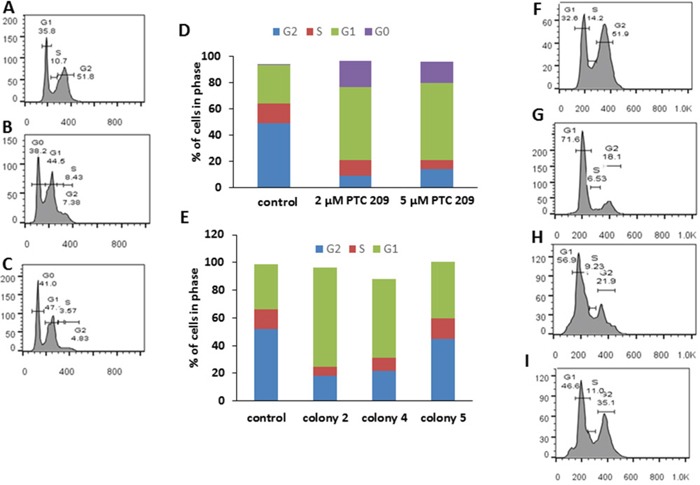
Bmi1 downregulation causes cell cycle arrest **(A)** Untreated (control) FMMC 419II cells. **(B)** Cells treated with 2 μM PTC 209. **(C)** Cells treated with 5 μM PTC 209. **(D)** Results of PTC 209 treatment shown as bar graphs. There is a G0/G1 cell cycle arrest in FMMC 419II cells treated with PTC 209 when compared to untreated cells. Similarly, FMMC 419II cells that have been transfected with a *Bmi1* shRNA show a G1 arrest. **(E)** Bar graphs of cell cycle profiles for FMMC 419II cells from control **(F)**, colony 2 **(G)**, colony 4 **(H)**, and colony 5 **(I)**. Cells stained with PI/RNAse staining buffer were run on a FACSAria flow cytometer and cell cycle progression was analyzed and quantified **(D, E)** using FlowJo.

Bmi1 inhibits expression of the Cdkn2a locus and therefore reduces the expression of the p16^Ink4a^ and p19^ARF^ proteins that negatively regulate cell cycle progression [13]. Flow cytometry shows inhibition of cell cycle progression in PTC 209 treated FMMC cells and in cells from colonies 2, 4, and 5. There is a marked decrease in the number of cells that are in the G2 phase of cell cycle in the treated cells (9.4%, 2 μM PTC 209) and (14.1%, 5 μM PTC 209) compared to the untreated cells (49.4%), with more cells arrested at the G0/G1 phase in the treated cells (2 μM PTC 209: 19.6% G0; 55.9% G1, 5 μM PTC 209: 16.3% G0 and 59.8% G1) than in the untreated cells (0.3% G0 and 29.6% G1) (Figure 2A-2D). Cells from colonies 2, 4, and 5 also display a similar cell cycle pattern with more cells arrested at the G1 stage, a phenotype associated with reduced Bmi1 expression (G1 phase: control, 32.6%; colony 2, 71.6%; colony 4, 56.9%; colony 5, 46.6%) (Figure 2E-2I and Supplementary Table 1).

We also observed changes in proliferation in the test cells in comparison to the control cells in a 48 hour MTT assay. Cells that are either treated with PTC 209 or transfected with Bmi1 shRNAs have a higher number of cells arrested at the G0/G1 stage than untreated cells (Supplementary Figure 1).

### Decrease in Bmi1 reduces mammosphere formation

**Figure 3:**
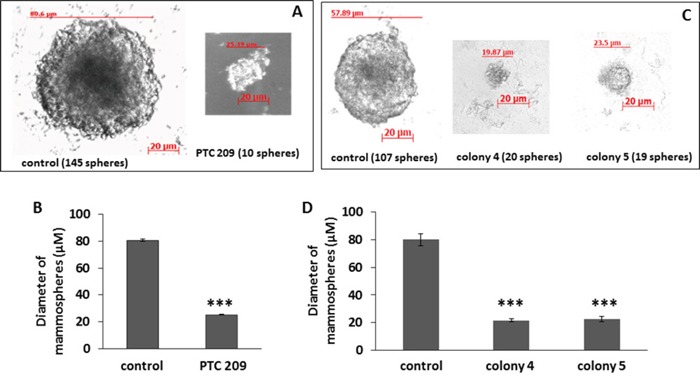
FMMC 419II cells treated with 2 μM PTC 209 or transfected with *Bmi1* shRNA display significantly lower mammosphere formation potential Treated cells were plated at a concentration of 1 cell/μl in an ultra-low attachment 96-well plate in serum free conditions. Phase contrast images at 20X magnification were taken of mammospheres (“spheres”) that were formed after a 2 week incubation **(A, C)**. The graphs **(B, D)** represent the mean ± S.E.M. of the mammosphere diameters. ***P <0.005.

The potential to from tumorspheres, or mammospheres in the case of breast cancer, is indicative of self-renewal of CSCs [34]. The effect of Bmi1 downregulation on self-renewal was assessed by the ability of a single cell to form a mammosphere when cultured in non-adherent conditions in serum-free media. PTC 209 treated cells (Figure 3A, 3B) and cells from colonies 4 and 5 (Figure 3C, 3D) form fewer mammospheres than the control cells (Supplementary Table 2), and the formed mammospheres are much smaller. Thus blocking Bmi1 expression inhibits the self-renewal property of the FMMC 419II CSC-like population.

### Flow cytometry analysis for expression of CD24 and CD49f

**Figure 4:**
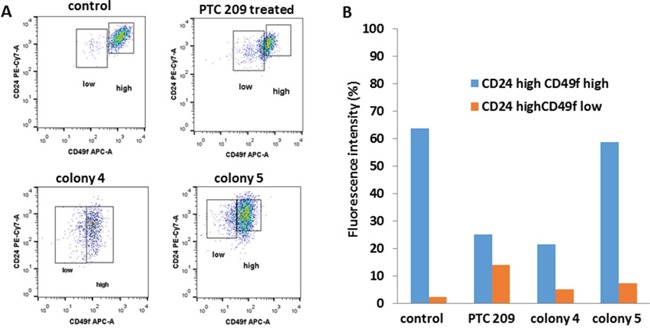
Flow cytometry analysis of CD49f and CD24 expression for FMCC 419II cells treated with 2 μM PTC 209 versus Bmi1 shRNA transfected colonies 4 and 5 were carried out **(A)**, and the fluorescent intensities were quantitated **(B)**.

Flow cytometry analysis showed that on treatment with 2 μM PTC 209, the FMMC cells showed a decrease in cells with high CD49f expression (Figure 4A and 4B). Similarly, cells from the Bmi1 shRNA transfected colonies, colony 4 and colony 5, showed lower CD49f expression than un-transfected control cells (Figure 4A and 4B).

### Reduced Bmi1 expression inhibits tumorigenicity *in vivo*

**Figure 5:**
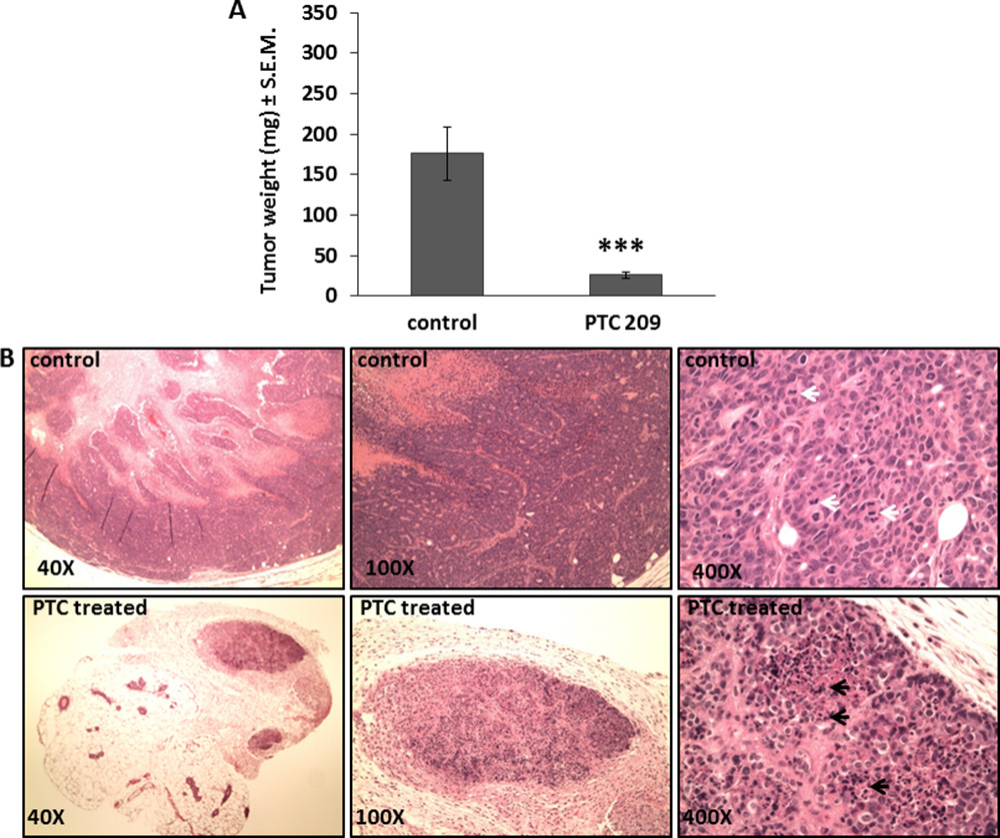
PTC 209 treatment results in decrease of tumor growth of FMMC 419II tumors **(A)** Tumors resected from the right and left 4^th^ mammary pad 4 weeks after implantation of 0.75×10^6^ FMMC 419II cells mixed with 50 μg PTC 209 were weighed and results are represented as the average weight (mg) ± S.E.M., ***P <0.005, n=6. **(B)** H and E staining of 6 μM tumor tissue sections were imaged at 40X, 100X, and 400X magnifications. Representative images from untreated (control) and PTC 209 treated tumors are shown. White arrows denote mitosis, black arrows denote apoptosis.

To explore the effect of Bmi1 downregulation on breast CSC growth *in vivo*, we determined the effect of treating the FMMC 419II cells with PTC 209 when they were transplanted into female FVB animals. Tumors generated from mice that received cells mixed with PTC 209 are uniformly much smaller than control tumors and weigh significantly less than tumors generated from untreated cells (26±4 mg versus 176±33 mg, respectively, Figure 5A). Histological analysis of tumors derived from PTC 209 treated cells show fewer mitotic cells and more apoptotic cells than do transplanted tumors from untreated cells. The untreated control tumor sections also have areas that follow the normal tumor growth pattern with central coagulative necrosis, whereas the treated tumors have larger areas of apoptotic cells (Figure 5B).

**Figure 6:**
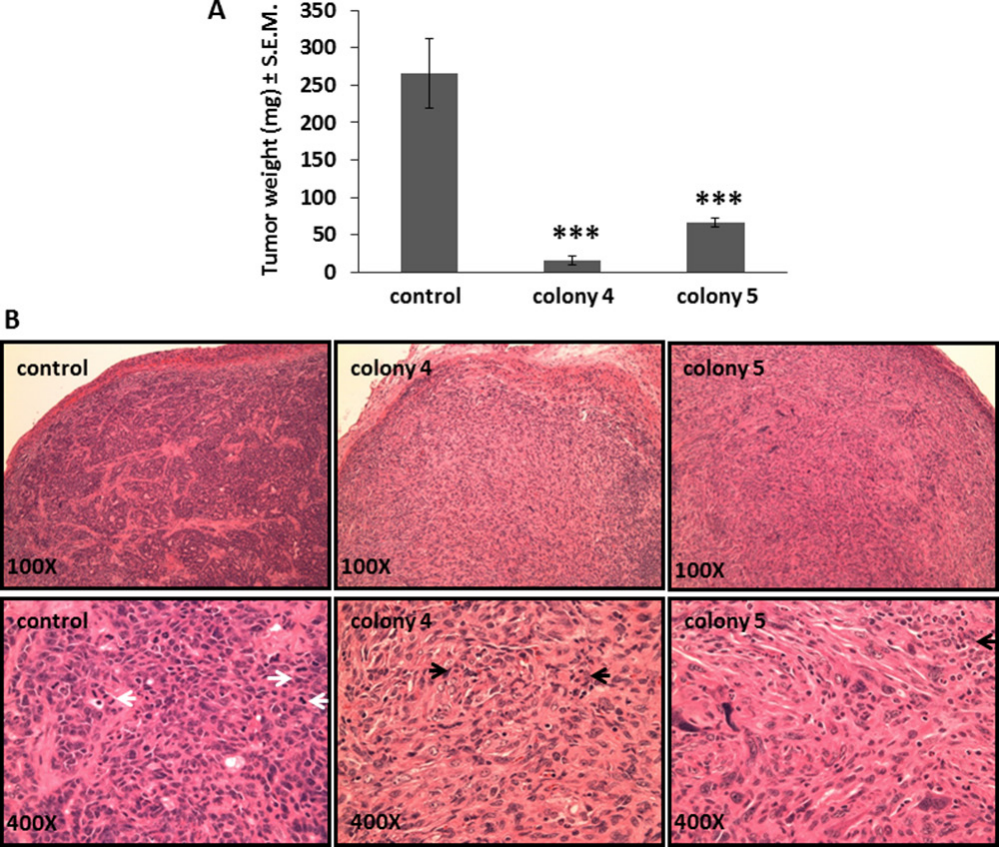
Bmi1 inhibition in FMMC 419II cells stably transfected with *Bmi1* shRNA causes reduced tumor growth when transplanted *in vivo* Tumors were collected 4 weeks after implantation from the 4^th^ mammary pad of FVB animals transplanted with 0.75×10^6^ control or transfected cells (colony 4 and colony 5) and weighed. **(A)** Tumor weights are represented as average (mg) ± S.E.M., ***P <0.005, n=5. **(B)** 6 μM tissue sections of tumor tissue were stained for H and E and imaged at 100X and 400X magnifications. Representative images of tumor derived from control, colony 4, and colony 5 cells are shown. White arrows denote mitosis, black arrows denote apoptosis.

Similarly, transplanted tumors derived from cells stably transfected with Bmi1 are significantly smaller and weigh less (16±6 mg and 67±6 mg) than untreated (control) tumors (265±46 mg) (Figure 6A). Tumor sites transplanted with untreated cells display dense growth of undifferentiated tumor cells with many actively dividing mitotic cells. Sites from tumors implanted with transfected cells have collections of fibroblasts and giant cells consistent with granulomas, with very few tumor cells (Figure 6B).

### Inhibition of Bmi1 reduces CD49f expression on the cell surface of breast CSCs

**Figure 7:**
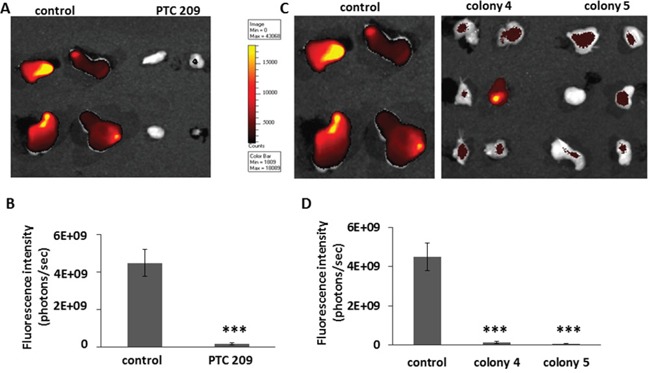
PTC 209 treated tumors with decreased Bmi1 expression show significantly lower expression of CSC marker CD49f **(A)** Tumors derived from transplantation of FMMC 419II cells treated with PTC 209, or **(C)** from colony 4 and colony 5 were imaged for CD49f fluorescence. The probe was an empty PLGA nano shell coated with anti-mouse CD49f antibody and conjugated to a Cy7 fluorescent molecule that was injected into the animals 24 hours before tumors were extracted. **(B, D)** Fluorescence intensity was calculated as photons emitted per second and graphed as average ± S.E.M., ***P <0.005.

A previous study established that the subpopulation of FMMC 419II cells that initiated tumors had a high expression of the cell surface molecule CD49f [7]. CD49f is a cell surface integrin that is associated with self-renewal properties in breast CSCs [35], and its elevated expression correlates with reduced survival of breast cancer patients [36]. We therefore tested the expression levels of CD49f in the tumors that were resected from the control and experimental groups of animals. PLGA NPs coated with anti-CD49f and Cy7 were injected into the animals 24 hours before tumors were collected, and fluorescence levels were measured using an IVIS imager. As expected, tumors from control animals showed high expression of CD49f. In striking contrast, tumors derived from cells treated with PTC 209 showed little or no expression of CD49f (Figure 7). Similarly, the tumors obtained from colonies 4 and 5 showed significantly less CD49f expression. Analysis with flow cytometry of cells treated *in vitro* with PTC 209 also shows a 60.37% decrease in CD49f expression, and there is a 66.14% decrease in CD49f expression in colony 4 and a 7.68% decrease in colony 5 as compared to untreated 419II cells. Thus, inhibition of Bmi1 led to a decrease in CD49f expression and potentially reduced self-renewal capabilities.

### Anti-cancer effects of systemic treatment with PTC 209

**Figure 8:**
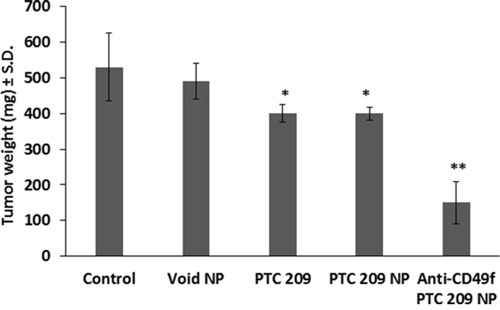
Effect of daily systemic treatment of PTC 209 in different forms on tumor growth Syngeneic mice were implanted in the 4th mammary pad of FVB animals with mouse CSC, FMMC 419II at 0.75×106 cells per implant. Animals were treated daily at 1 mg/Kg, subcutaneously, with free PTC 209, PLGA-PEG encapsulating PTC 209 (PTC 209 NP), or PLGA-PEG conjugated with anti-CD49f and encapsulating PTC 209 (Anti-CD49f PTC 209 NP) as compared to vehicle control or void PLGA-PEG NPs. Data represent mean ± S.D., n = 5/group, *P <0.05 versus control or void NPs, ** P <0.01 versus all arms.

Treatment of syngeneic mice bearing transplanted FMMC 419II tumors with anti-CD49f coated NPs containing PTC 209 resulted in reduction in tumor weight and size not observed in mice receiving other NP formulations. Tumor weights in each group (n=5) are shown in Figure 8. Nano-targeted delivery of PTC 209 (PLGA-PEG conjugated with anti-CD49f and encapsulating PTC 209) administered daily at 1 mg/Kg, subcutaneously for 2 weeks, demonstrated distinct suppression of breast tumor growth (**P <0.01) as compared to free PTC 209 or PLGA-PEG NPs encapsulated with PTC209 (Figure 8).

## DISCUSSION

Our results indicate that Bmi1 plays a functional role in regulation of CSCs in breast cancer. To downregulate Bmi1 we used two parallel and independent strategies: a small molecule Bmi1 inhibitor and a transfection of *Bmi1*-specific shRNA plasmid. Results of these experiments documented significant inhibition of the Bmi1 mRNA and protein expression in CSCs and showed that inhibition of Bmi1 expression in these cells abrogates stem cell-specific properties and significantly reduces tumor growth in animals.

Bmi1 downregulation led to a G0/G1 arrest during cell cycle progression and a growth inhibition of CSCs. Colonies 2 and 4 were more effective in affecting the cell cycle as compared to colony 5, while all colonies exhibited comparable suppression of Bmi1 expression; the exact reason for the differences is not clear. However, PTC 209 showed effective suppression of Bmi1 expression and cell cycle effects. These results are highly consistent with the previously reported mechanisms of biological activity of the Bmi1 protein. Bmi1 acts as a positive regulator of cell cycle progression by elimination of the P16/p14 cell cycle check points [37]. Bmi1 causes the active repression of the cyclin kinase inhibitors (CKI) p16INK4a and p19ARF proteins [38]. The decreased expression of Bmi1 in the tumor stem cells relieves this repression, thus causing increased expression of the negative regulators of cell cycle progression. Bmi1 has also been implicated in regulation of the PI3K/AKT pathways involved in cancer cell progression [16].

It is generally accepted that mammosphere formation assays help identify and characterize breast CSCs. Dontu *et al*. first showed that mammary epithelial cells cultivated and propagated in non-adherent and serum free conditions consist of a core population of undifferentiated cells that recapitulate the properties of stem cells [39]. Mammosphere formation was later employed to identify breast CSCs in a number of studies [40]. We show that Bmi1 is necessary for mammosphere formation and thereby is essential for self-renewal of the mammary CSCs. PTC 209 treated cells and cells derived from the stable transfection colonies show a decrease in both the percentage of mammospheres formed as well as the diameter of the spheres formed. Thus inhibition of Bmi1 expression in these CSCs causes lower potential of self-renewal and decreases clonogenecity compared to untreated (control) cells.

Kreso *et al*. first characterized PTC 209 as a pharmacological inhibitor of Bmi1 in a colorectal cancer model and described the loss of stemness and growth inhibition *in vitro* of human colorectal CSCs upon PTC 209 treatment, as well as inhibition of tumor growth *in vivo* when nude mice with tumor cell xenografts were treated with the Bmi1 inhibitor [20]. We show that implantation of mammary CSCs mixed with a one-time dose of the inhibitor causes a significant decrease in tumor growth, which is likely caused by the inhibition of tumor initiation. Histological analysis of the tumor mass and surrounding tissue indicates that the treatment with the Bmi1 inhibitor results in increased apoptosis and inhibition of mitoses. We note the areas in control tumor masses that are clear of cancer cells show a pattern of coagulative necrosis, which is the typical growth to death pattern of cancer cells as they grow away from the blood supply. Tumors generated by cells from the stable transfection colonies are significantly smaller than the control tumors. The large areas of granuloma present in the tumor masses might indicate the early clearance of the weakly tumorigenic cancer cells and subsequent wound healing. It should be noted that these experiments were short-term with the tumors being resected out of the animals within 4 weeks of implantation. We plan to follow tumor growth for a longer time to better elucidate the effect of Bmi1 on growth and progression of the mammary tumors. This study proves however, that inhibition of just Bmi1 alone is sufficient to lower the tumor initiation capabilities of the mouse mammary CSCs.

CD49f is a cell surface integrin that was initially used as a biomarker to identify CSCs from the MMTV-PyMT primary tumors. CD49f has been implicated in self-renewal and tumorsphere formation in breast cancer cells [41], and high expression of CD49f positive breast cancer cells show higher tumorigenicity [42], higher metastatic potential [43], and reduced survival [36] compared to CD49f negative cells. The reduced CD49f expression seen both *in vitro* and *in vivo* as a result of Bmi1 inhibition suggests that Bmi1 may regulate CD49f expression and function in CSCs. In our mouse mammary stem cell model, CD49f therefore has a dual role: as a CSC identifying moiety to specifically target CSCs and as a functional predictor of the relative strength of the stemness potential in these cells. Bmi1 is a transcriptional repressor and regulates multiple downstream targets both directly and indirectly [44]. Whereas the transcriptional link between Bmi1 and CD49f has not been elucidated, there is evidence that Bmi1-mediated upregulation of Sox2 [45] may maintain expression of CD49f and thereby, self-renewal and tumorigenicity [46].

An important advantage of using FMMC 419II cells is the ability of these cells to grow in an immunocompetent mouse without the risk of rejection. The FVB strain of mice is syngeneic to the MMTV-PyMT mice from which the FMMC 419II cells were originally derived and therefore offers an *in vivo* model system that is physiologically more relevant than tumor transplantation into immunodeficient mice.

Many studies have focused on Bmi1 expression and function in primary breast cancer cells from patients, or human breast cancer cell lines, and determined that loss of Bmi1 leads to loss of characteristics associated with CSCs. PTC 209 reduced Bmi1 mRNA *in vitro* at 2-5 μM by ~ 50%, reflecting its potency, but this does not suggest its effect to be direct or indirect. For proof of the concept, we have used molecular approaches to illustrate the impact of Bmi1 downregulation on breast CSC in a novel model of mouse immunocompetent breast CSC-driven breast tumor generation. Additionally, we tested a nano-targeting strategy using anti-CD49f conjugated to PLGA-PEG NPs encapsulated with PTC 209 to illustrate an effective strategy in suppressing breast CSC derived tumor growth.

The present contribution is the first study to directly assess the effect of Bmi1 inhibition in a highly enriched population of CSCs *in vitro* and after transplantation *in vivo* into syngeneic fully immunocompetent animals. We believe this is critically important given the central role of the *Bmi1* gene in the maintenance of stemness state in both normal and malignant stem cells. We have shown through both pharmacological inhibition and by genetic silencing that Bmi1 is implicated in growth and maintenance of stemness of these mammary CSCs. Unfortunately, systemic administration of PTC 209 after tumor implantation resulted in early mortality of the animals, even when doses were reduced to 15-fold less than what was used in the study by Kreso *et al*. [20] (data not shown). The reasons for this unexpected toxicity *in vivo* of the PTC 209 remain unknown. Therefore, we used anti-CD49f conjugated NPs containing PTC 209 to deliver the Bmi1 inhibitor directly to CSCs at lower doses of PTC 209 where free PTC 209 was safe but ineffective as compared to the anti-CD49f conjugated NPs containing PTC 209.

In our study, PTC 209 administered systemically illustrated major toxicity in mice, which was not discussed in the previous report by Kreso *et al*. [20]. For initial proof of the concept for the role of Bmi1, we treated the mouse breast CSC line FMMC 419II with PTC 209 and implanted in mice versus implanting FMMC 419II stably transfected with Bmi1 plasmid. Data showed comparable efficacy in all *in vitro* and *in vivo* assay and model systems used. We did not do a clearance study for PTC 209 from the tumor but rather compared local treatment of FMMC 419II cells with PTC 209 as compared to FMMC 419II stably transfected with Bmi1 plasmid where comparable data were shown. Intra-tumoral implant of FMMC 419II stably transfected with Bmi1 plasmid in Matrigel versus FMMC 419II cells treated with PTC 209 demonstrated comparable efficacy. Further confirmations were provided by the systemic nano-targeted delivery of PTC 209 (1 mg/Kg, subcutaneously, daily for 2 weeks) where the anti-cancer efficacy versus the same dose of free PTC 209 was significantly (**P <0.01) improved.

## MATERIALS AND METHODS

A more detailed Materials and Methods is provided in the Supplementary Materials and includes quantitative PCR, flow cytometry, cell cycle analysis, cell proliferation and viability assay, mammosphere formation assay, NPs' synthesis and characterization, *in vitro* (IVIS) imaging, and histology.

### Cells and cell culture

Cell culture reagents such as Dubelco's Modified Eagle Medium (DMEM), fetal bovine serum (FBS), trypsin-EDTA, penicillin/streptomycin and glutamate were purchased from Sigma Aldrich (St. Louis, MO). The CSC-enriched FMMC 419II mouse mammary tumor cells were isolated from primary tumors in MMTV-PyMT transgenic mice and characterized with flow cytometry and sorting in the lab of S.S. Cells were cultured in DMEM supplemented with 10% FBS and penicillin/streptomycin (1%) and grown to sub-confluency at 37°C in a humidified atmosphere of 5% CO_2_/95% air, then sub-cultivated and expanded for experimental use. For treatment with the Bmi1 small molecule inhibitor PTC 209 (Xcess Biosciences, San Diego, CA), cells were trypsinized, washed, and seeded into 6-well plates at around 30% confluency. Twenty-four hours later, appropriate concentrations of PTC 209 were added to the cells and incubated for 48 hours. After treatment, cells were trypsinized, washed, and equal number of viable cells from the PTC 209 treated and DMSO control wells were used for further experiments.

### shRNA transfection

Mouse *Bmi1* shRNA plasmid (Santa Cruz Biotechnology, Dallas, TX) consists of 3 target-specific lentivirus vector plasmids to knock down Bmi1 expression along with a puromycin selection gene. FMMC cells were transfected according to the manufacturer's protocol using transfection support reagents (Santa Cruz Biotechnology). Briefly, cells were grown in 6-well culture plates until they were 60% confluent. They were then transfected with the *Bmi1* shRNA plasmid in the transfection medium provided in the absence of serum or antibiotics and incubated at 37°C. Five to 7 hours later, DMEM supplemented with 20% FBS and 2 μg/ml puromycin was added and cells were further incubated per the manufacturer's protocol. Cells were then cultured and maintained in DMEM supplemented with 10% FBS and 1 μg/ml puromycin until the formation of transfected colonies appeared. The colonies were spot trypsinized using small spherical discs of sterile filter paper, and the discs were transferred to new culture plates for expansion of the transfected colonies. Three colonies were identified and named as colony 2, colony 4, and colony 5. Knockdown of Bmi1 expression was confirmed from these expanded colonies with PCR and western blot analysis.

### Flow cytometry analysis for expression of CD24 and CD49f

FMMC cells were counted, plated at 1 × 10^6^ cells/plate and treated with 2 μM PTC 209 for 48 hours. Cells were then washed, trypsinized, and incubated with fluorescent antibodies against CD24 and CD49f. Expression of these proteins was then determined with flow cytometry.

### Nanoparticles synthesis and characterization

PLGA-PEG NPs encapsulating PTC 209 were synthesized by a solvent diffusion method, previously described by our group [26–28]. Details are included in the Supplementary.

### *In vivo* animal studies

Six to 8 week old female wild type FVB mice (Harlan Laboratories, Indianapolis, IN) were used for the *in vivo* experiments. FVB mice are syngeneic to MMTV-PyMT transgenic mice, from which the FMMC 419II tumor cells were derived. Mice were maintained at the animal facility of the Albany Stratton Veterans Affairs Medical Center (VAMC), Albany, NY, under an animal protocol approved by the VAMC Institutional Animal Care and Use Committee. Mice were allowed to acclimate for 5 days after arrival at the animal facility prior to experimental treatments and were housed under conditions of a 12 hour light/dark cycle, 20−24°C, and 60-70% humidity. Food and water were provided ad libitum. We mixed 0.75×10^6^ FMMC cells with Matrigel alone or Matrigel and 50 μg PTC 209 and implanted the cells into the left and right 4^th^ mammary fat pad. Four weeks after implantation, tumors were excised and weighed and analyzed histologically. The CD49f^+^ CSCs in the tumor were identified by using a PLGA nano shell coated with anti-CD49f antibody and tagged with fluorescent Cy7. The tagged NPs were injected subcutaneously into the animals 13 days after cell implantation Twenty-four hours later, tumors were collected and the fluorescence intensity was measured using an *in vivo* imaging system (IVIS^®^, Xenogen, Toronto, ON, Canada) (Supplementary Figure 4). Photographic and fluorescent images were taken at constant exposure time. Signal intensity in regions of interest was analyzed using the Xenogen IVIS^®^ Living Image software version 3.2.

### Systemic treatment daily for 2 weeks

Systemic treatment was with PLGA-PEG NPs conjugated with anti-CD49f and encapsulating PTC 209 versus free PTC 209, or PLGA-PEG NPs encapsulating PTC 209 and as compared to control and void PLGA-PEG NPs. In all arms having PTC 209, it was dosed at 1 mg/Kg, subcutaneously, daily for 2 weeks.

### Statistics

Results are presented as mean ± standard error means (S.E.M.) or ± standard deviation (S.D.) as given in Figure legends. Comparisons between control and experimental groups were statistically analyzed using students t-test and ANOVA using SigmaPlot 10.0 software. Differences between control and experimental end points were considered statistically significant if P <0.05.

## SUPPLEMENTARY MATERIALS FIGURES AND TABLES



## Abbreviations

ALDH1: aldehyde dehydrogenase 1
CSC: cancer stem cell
NP: nanoparticle
PLGA-PEG NPs: poly (lactic-*co*-glycolic acid)poly (ethylene glycol) nanoparticles.
