# Supplementary material for: Downregulation of Bmi1 in breast cancer stem cells suppresses tumor growth and proliferation
Source: Oncotarget. 2017 Mar 17;8(24):38731–42. doi: 10.18632/oncotarget.16317 (PMC5503567; doi:10.18632/oncotarget.16317)
Supplement: Supplementary file 1 [file oncotarget-08-38731-s001.pdf]

Downregulation of Bmi1 in breast cancer stem cells suppresses tumor growth and proliferation

SUPPLEMENTARY DATA

Quantitative PCR

mRNA was purified from cell using the Qiagen RNEasy mini kit (Qiagen, Valencia, CA) and quantified using the NanoDrop 2000. Two µg of RNA was then reverse transcribed into cDNA using the Qiagen QuantiTect Reverse Transcription kit. *Bmi1* and *GAPDH* mRNA expression levels were then quantified with quantitative real time PCR using a Taqman gene expression assay with primer/probe sets specific to *Bmi1* (FAM probe, Mm03053308_m1) and *GAPDH* (VIC probe, Mm99999915_g1) from Life Technologies Corporation (Grand Island, NY). Primer/probe sets to both *Bmi1* and the internal control gene *GAPDH* were added together to the cDNA samples along with the Kapa Probe Fast qPCR kit (Kapa Biosystems, Wilmington, MA), plated in a 96-well plate and run on a Mastercycler® RealPlex² from Eppendorf. The reduction in gene expression of *Bmi1* due to PTC 209 treatment or by *Bmi1* shRNA transfection was calculated using C_T (threshold cycle) values and the comparative C_T method [1].

Relative expression of *Bmi1* was expressed as a comparison in C_T values of Bmi1 between the PTC 209 treated or the Bmi1 shRNA transfected sample and the untreated or the control sample, relative to the C_T of the internal control gene *GAPDH*. The fold change was calculated according to the equation:

Fold change = $2^{-\Delta\Delta}{}_{CT}$

where $\Delta\Delta C_T = [(C_T\ Bmi1 - C_T\ GAPDH)$ treated - $(C_T\ Bmi1 - C_T\ GAPDH)$ control].

The percentage reduction in gene expression of Bmi1 due to PTC 209 treatment or by *Bmi1* shRNA transfection was expressed as 100% / fold change.

Flow cytometry

Cells were trypsinized, washed, and suspended at 1 x 10^6 cells/ml for flow cytometry analysis. Cells were resuspended in PBS+1% BSA and stained with appropriate concentrations of CD24-PE-Cy7 (Phycoerithrin-Cyanine7) and CD49f-AF647 (Alexa Fluor 647) (BioLegend, San Diego, CA) for 30 minutes on ice. The stained cells were run on a BD FACSAria flow cytometer and data was analyzed using the FACSDIVA software (BD Biosciences, San Jose, CA).

Cell cycle analysis

Cells, treated with various concentrations of PTC 209 for 48 hours, were washed and then cultured for another 24 hours to synch up the growth cycle. *Bmi1* shRNA transfected cells were trypsinized and washed in PBS. Cells were then centrifuged, fixed, and permeablized by slow addition of ice-cold methanol to the pellet under constant vortexing and incubation at 37°C for 20 minutes. Cells were then washed in PBS+0.1% BSA and centrifuged at 200 x *g*. Cell pellets were stained with PI/RNAse staining buffer (BD Biosciences) and run on the FACSAria flow cytometer. Doublets were excluded and the DNA content of the single cells was analyzed to obtain the percentage of cells in each phase of the cell cycle (Supplementary Table 1). Analysis of the data was done using FlowJo VX.0.7 data analysis software (FlowJo, LLC, Ashland, OR).

Cell proliferation and viability assay

Proliferation and viability were measured using the Vybrant MTT (3-(4, 5-dimethylthiazol-2-yl)-2, 5-diphenyltetrazolium bromide) Cell Proliferation kit from Thermo Fisher Scientific. Cells treated with 2 µM and 5 µM PTC 209 or *Bmi1* shRNA transfected cells were trypsinized and washed with media. Cells were then counted, plated at appropriate concentrations in 96-well plates, and incubated at 37°C for 48 hours. After incubation, MTT assay was performed using the manufacturer's protocol. Briefly, the MTT reagent was added to each well at 0.5 mg/ml and incubated for 3 hours. The formazan crystals were then solubilized with DMSO and the absorbance read at 540 nm as an indicator of cell growth and proliferation (Supplementary Figure 1). Each sample was plated in triplicate and results are displayed as mean ± S.E.M.

Mammosphere formation assay

The mammosphere formation assay was performed according to Lo *et al.* [2]. Mammosphere culture medium was prepared by addition of 20 ng/ml EGF, 10 ng/ml basic FGF, 5 µg/ml insulin, and 0.4% bovine serum albumin to DMEM. Cells were resuspended in this medium at a concentration of 1 cell/µl and 200 µl was seeded into 10 wells of an ultra-low attachment 96-well plate. The top and bottom rows were filled with 200 µl of 1X PBS and the plates were sealed with laboratory tape to prevent evaporation. Plates were incubated at 37°C for 2 weeks. After incubation, tumorspheres were counted under a phase-contrast microscope at 20X magnification

(Supplementary Table 2) and the diameters of the tumorspheres were also measured.

Nanoparticles (NPs) synthesis

Briefly, 100 µl of PLGA-PEG and maleimide-PLGA-PEG in a ratio of 9:1 (80 mg/ml) were mixed with 20 µl of PTC 209 (20 mg/ml in DMSO). This solution was added drop by drop to 10 ml of 1% PVA solution under constant magnetic stirring. It was then sonicated in a probe sonicator for 30 seconds and stirred for another 2 hours. Finally, the solution was dialyzed to remove free PTC 209 and residual DMSO. Anti-CD49f was conjugated to the surface of the PTC 209-encapsulated NPs by first thiolating the anti-CD49f using Traut's reagent. The thiolated anti-CD49f readily reacts with the maleimide group on the NPs (Supplementary Figure 2). This nanoformulation was lyophilized in the presence of cryoprotectant 1-2% sucrose or mannitol to get it in a powdered form and then was re-dispersed in DI water for further studies.

Dye-conjugated nanoparticles (NPs)

To facilitate *in vivo* imaging to determine cellular uptake, the NPs were labeled with a Cy7-NHS dye as per our previously described method [3] to attach an amine arm to the terminal COOH of the NPs.

Dynamic laser light scattering (DLS)

The size distribution of PLGA-PEG NPs in aqueous dispersions was determined using a Malvern zeta sizer (Malvern Co, Westborough, MA, USA). We observed an average size of 225 nm in diameter (Supplementary Figure 3).

Entrapment efficiency (EE)

EE of the PLGA-PEG NPs encapsulating docetaxel was determined by filtering a known amount of PLGA-PEG NPs through a 100 kDa filter membrane to separate the free PTC 209 from the encapsulated PTC 209. We calculated entrapment (loading) efficiency (EE%) for PTC 209 using the values for the total concentration of PTC 209 in the system (free + encapsulated, $[D]_0$) and the PTC 209 encapsulated in the NPs, ($[D]_f$) calculated using the equation EE$\% = [D]_f / [D]_0 \times 100$. We obtained a value of EE% of ~70%.

IVIS imaging

In vivo imaging with Cy7-labeled anti-CD49f nanoparticles

Anti-CD49f NPs or unconjugated NPs were labeled with Cy7 and injected subcutaneously (data not shown, essentially the same results as IP) or IP (Supplementary Figure 4) into normal FVB mice or FVB mice with tumors produced by injection of 1 x 10^5 FMMC 491II cells in 50% Matrigel in the lower mammary fat pads. IVIS imaging was done 1 week after injection of the cells when tumors were very small (less than 3 X 3 X 2 mm). IVIS images were taken before injection, immediately after injection and 1, 2, 3, 4 and 24 hours after injection. Tumors were then harvested to study the fluorescence intensity. With either NP injection site, there is immediate localization of both the NP Cy7 and anti-CD49f Cy7 NPs to the tumor sites. However, there is much less and transient (less than 4 hours) labeling of the tumor site with NP Cy7 compared with the heavier and longer lasting (over 24 hours) labeling using anti-CD49f-NP Cy7. There is no prolonged labeling of any other tissue site. These data support the hypothesis that the anti-CD49f coated NPs are selectively localized to tumor tissues and not to any normal tissues.

Histology

Tumor tissues collected at termination were fixed in 4% paraformaldehyde for 4 hours and then transferred to distilled water. Tissues were then processed by paraffin embedding and were cut into 6 µm sections that were placed on microscopy slides. For H and E staining, slides were hydrated and then stained in hemtoxylin for 10 minutes. The stain was then developed by passing the slides through 3 acidified ethanol washes and 5 minute incubation in ammonia. Slides were then stained in eosin for 40 seconds and dehydrated by passing through 3 washes of 100% ethanol and 3 washes of xylene before coverslips were affixed. Tumor sections were then visualized using bright field microscopy at 10X and 20X magnification.

REFERENCES

1. Schmittgen TD, Livak KJ. Analyzing real-time PCR data by the comparative C(T) method. Nat Protoc. 2008; 3:1101-1108.
2. Lo PK, Kanojia D, Liu X, Singh UP, Berger FG, Wang Q, Chen H. CD49f and CD61 identify Her2/neu-induced mammary tumor-initiating cells that are potentially derived from luminal progenitors and maintained by the integrin-TGFbeta signaling. Oncogene. 2012; 31:2614-2626.
3. Bharali DJ, Sudha T, Cui H, Mian BM, Mousa SA. Anti-CD24 nano-targeted delivery of docetaxel for the treatment of prostate cancer. Nanomedicine. 2017; 13:263-273.

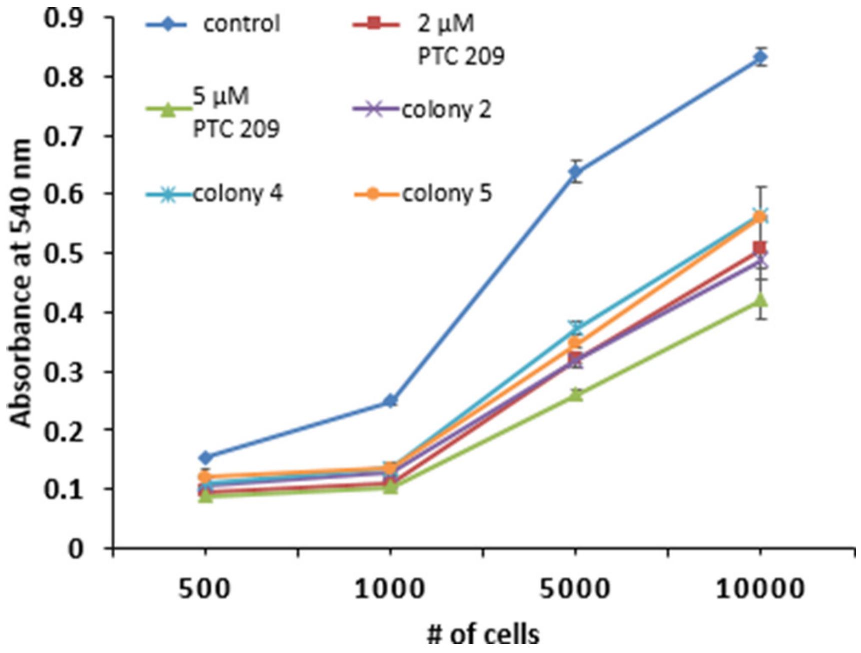

Supplementary Figure 1: Lower Bmi1 expression levels correlate with a decrease in the rate of proliferation of FMMC cells *in vitro*. After treatment, 500, 1000, 5000, and 10000 cells/well were grown for 48 hours and the proliferation measured with MTT assay. Cells were grown in triplicate, and results are presented as mean ± S.E.M.

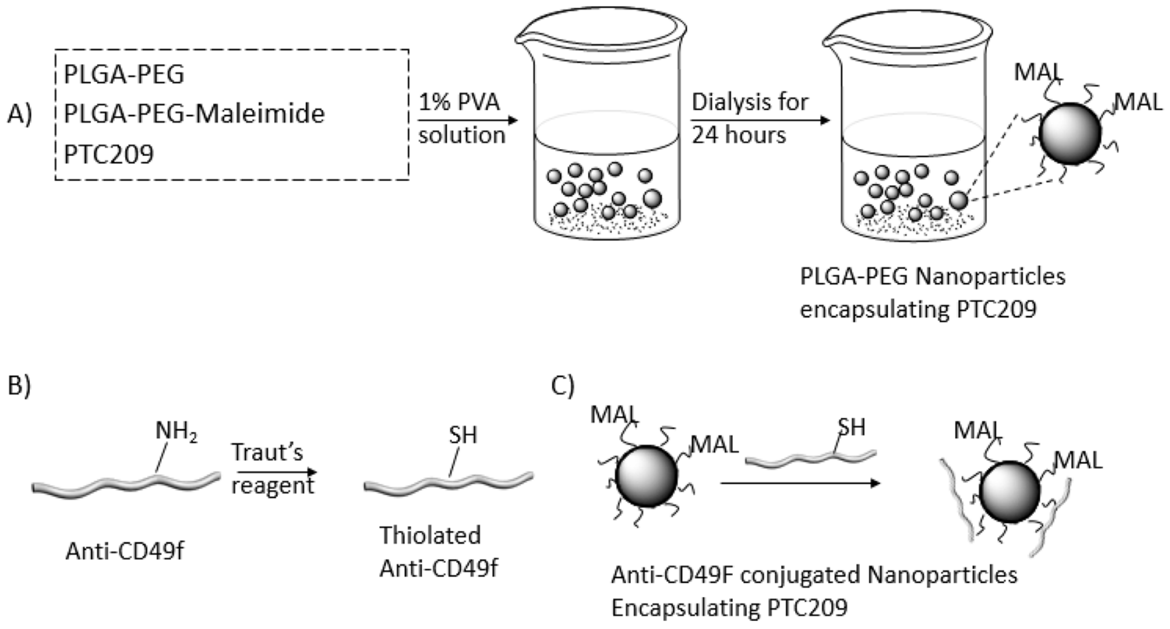

Supplementary Figure 2: Schematic diagram illustrating (A) the synthesis of PLGA-PEG nanoparticles encapsulated with PTC 209, (B, C) conjugation with anti-CD49f. Abbreviations: MAL, maleimide; PLGA, poly (lactic-co-glycolic acid); PEG, poly (ethylene glycol); PVA, poly vinyl alcohol.

			Size (d.nm):	% Intensity:	St Dev (d.nm):
Z-Average (d.nm):	225.2	Peak 1:	272.2	100.0	111.7
PdI:	0.203	Peak 2:	0.000	0.0	0.000
Intercept:	0.979	Peak 3:	0.000	0.0	0.000
Result quality :	Good				

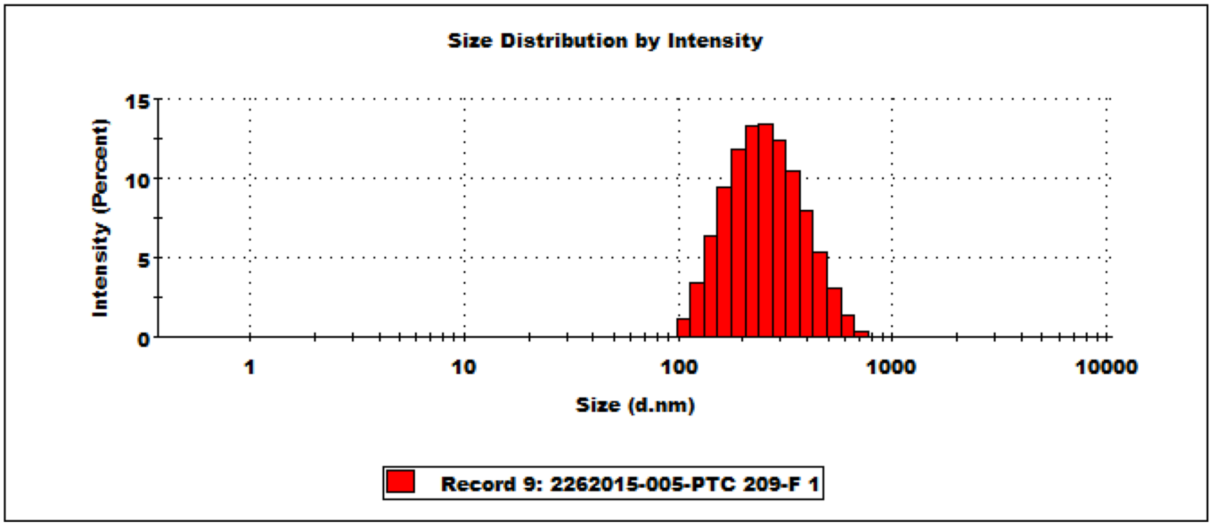

Supplementary Figure 3: Size measurement of nanoparticles encapsulating PTC 209. Average size was 225 nm in diameter, as measured with Dynamic Light Scattering (DLS).

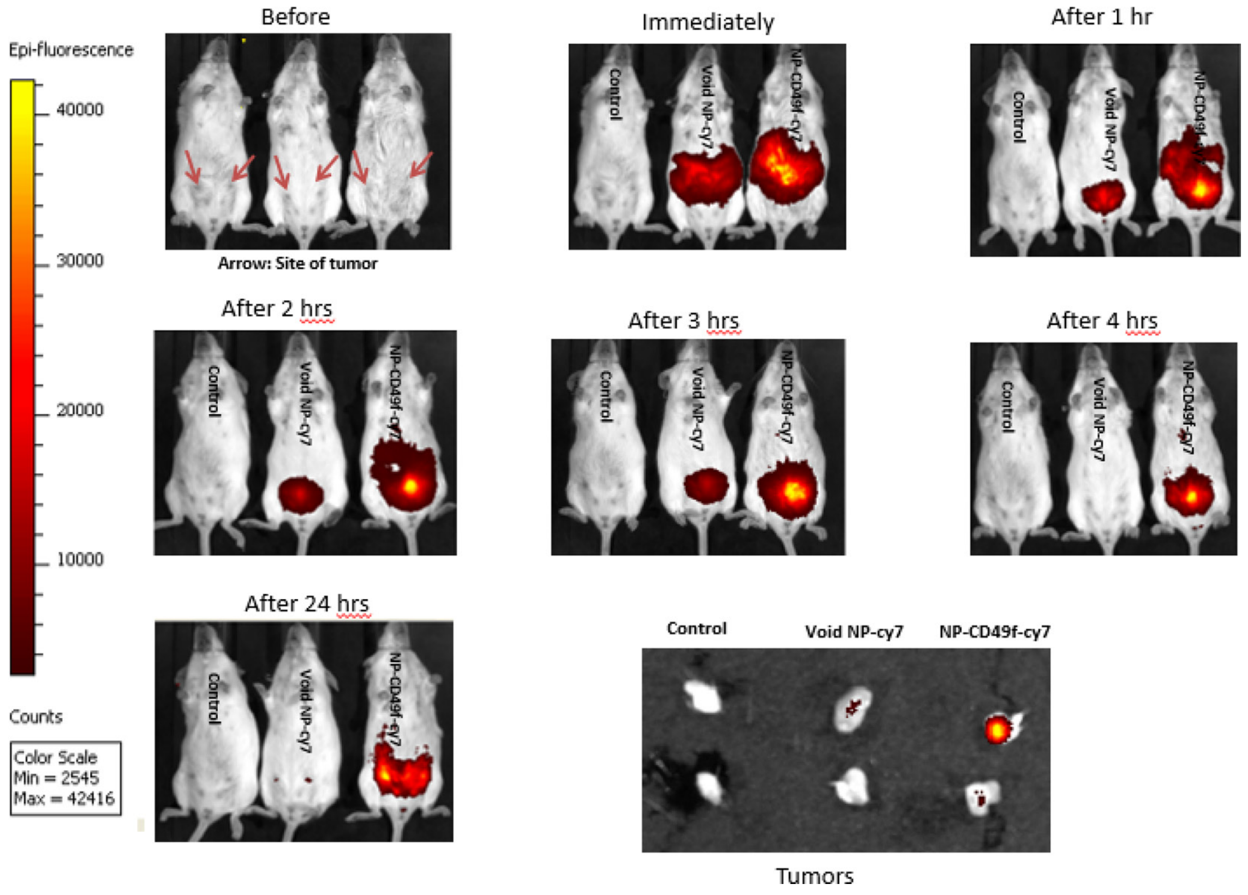

Supplementary Figure 4: ***In vivo*** **imaging (IVIS) of Cy7-labeled anti-CD49f conjugated PLGA nanoparticles over time in transplanted FMMC 419II breast cancer in mice.**

Supplementary Table 1: Percentage of cells in cell cycle phase

	G0	G1	S	G2
**Untreated**	0	35.8	10.7	51.8
**2 µM PTC 209**	38.2	44.5	8.43	7.38
**5 µM PTC 209**	41.0	47	3.57	4.83
**Control**	0	32.6	14.2	51.9
**Colony 2**	0	71.6	6.53	18.1
**Colony 4**	0	56.9	9.23	21.9
**Colony 5**	0	46.6	11.0	35.1

Supplementary Table 2: Percentage of mammospheres formed

	# Spheres/Cells	Percentage of mammospheres formed
**Untreated**	145/2000	7.25
**2 µM PTC 209**	10/2000	0.5
**Control**	107/2000	5.35
**Colony 4**	20/2000	1
**Colony 5**	19/2000	0.95
